# An Unusual Case of Bilateral Granulomatous Mastitis

**DOI:** 10.1155/2013/694697

**Published:** 2013-05-23

**Authors:** C. A. Pistolese, R. Di Trapano, V. Girardi, E. Costanzo, I. Di Poce, G. Simonetti

**Affiliations:** Department of Diagnostic Imaging, Molecular Imaging, Interventional Radiology and Radiation Therapy, University Hospital Tor Vergata, Viale Oxford, 81-00133 Rome, Italy

## Abstract

Idiopathic granulomatous mastitis (IGM) is an uncommon benign disorder of the breast. At clinical examination, IGM is characterized by an inflammatory process of the breast, usually unilateral. Possible clinical findings are palpable mass with erythematous skin, pain, sterile abscesses, fistula and nipple retraction. Mammography and ultrasound findings are not specific for IGM. Magnetic resonance imaging (MRI) is a useful tool for the differential diagnosis; it is also necessary to delineate the exact extension of the disease and to plan the correct treatment. Final diagnosis is histological. We described an unusual case of IGM with bilateral involvement in a patient with history of pacemaker implantation and IGM typical clinical symptoms. Mammography, ultrasound, and MRI examinations were performed to identify the inflammatory disorder and to plan the correct therapy. Imaging features were correlated with final histological diagnosis of IGM.

## 1. Introduction 

Idiopathic chronic granulomatous mastitis is a rare benign inflammatory breast disease.

In a study done by Baslaim et al. histopathology confirmed cases of IGM represented 1.8% of cases out of 1,106 women with benign breast diseases. The true prevalence of IGM is unknown.

IGM is an idiopathic condition. Mechanisms that have been proposed as etiologic factors include chemical reaction associated with oral contraceptive pills, pregnancy, breast feeding, autoimmune phenomenon, infection with yet unidentified pathogens, breast trauma, hyperprolactinemia with galactorrhea, and alpha-1-antitrypsin deficiency [1, 2].

IGM remains a diagnosis of exclusion, and the clinical findings are nonspecific [3]. 

Clinically, IGM presents as an inflammatory process of the breast that can mimic an inflammatory cancer or an abscess [4]. At initial presentation, the most common clinical finding is a palpable mass with erythematous skin changes [5, 6]. Other reported clinical symptoms include pain, sterile abscesses, fistula and nipple retraction. Unilateral involvement is typical; bilateral IGM is uncommon although it has been reported in the literature [7, 8]. 

Radiological findings are not specific for IGM. Mammographic and ultrasonographic most frequent findings are asymmetric diffuse increased density of fibroglandular tissue and hypoechoic mass lesions or nodular structures, respectively.

Breast magnetic resonance imaging (MRI) can be useful to characterize the lesions, but it is not diagnostic. 

Histopathological evaluation plays a crucial role in the diagnosis of IGM [9].

Treatment includes an initial conservative approach and an eventual secondary surgical resection, which are both associated with similar recurrence rates, on order of 25% [4]. 

We describe an unusual case of bilateral IGM, with an initial localization in the left breast, surgically treated, and a secondary extension to the contralateral breast. 

## 2. Case Presentation

A 49-year-old woman was referred to our institution with a 3-month history of severe pain in the left breast. 

A clinical examination of the breasts showed a diffused hardened and erythematous skin with multiple cutaneous ulcers in the left breast. 

The patient denied previous use of oral contraceptives, estrogens, recent breast trauma, and personal and familiar history of breast cancer and autoimmune pathology. She was not positive for fever.

The patient referred a pacemaker implantation on the left side few months before. 

**Figure 1:**
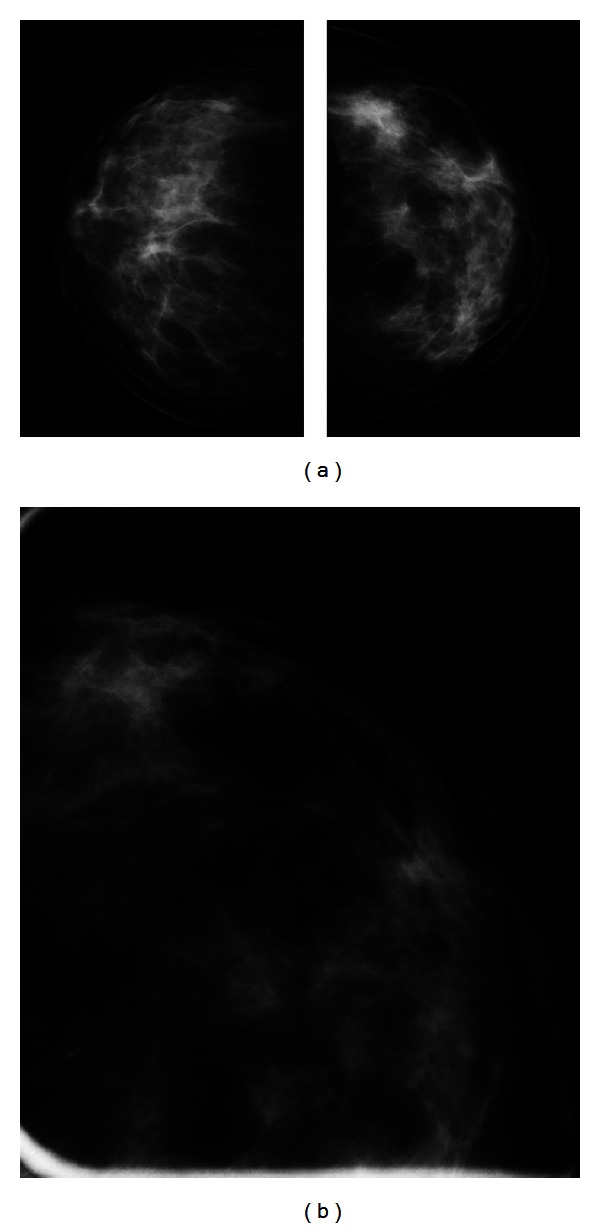
Bilateral craniocaudal projection (a) and magnification view (b) show an asymmetrical increased density in the lateral quadrants of the left breast.

A digital mammography, performed with GE Senographe DS (General Electric, Milwaukee, USA), using standard projections, revealed an asymmetrical density in the lateral quadrants of the left breast (Figure 1). 

**Figure 2:**
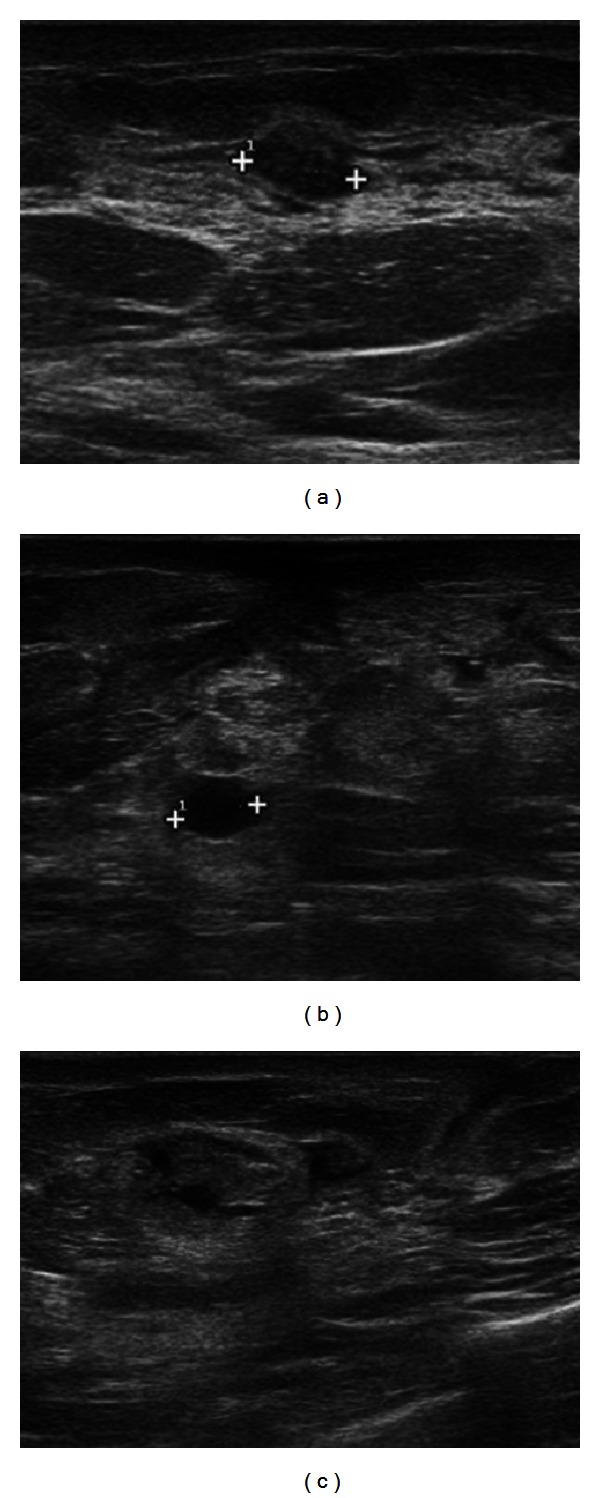
US shows hypoanechoic fluid confluent areas and in the upper outer quadrant (a) and in the lower outer quadrant (b and c) of the left breast and a diffused ipsilateral parenchymal and cutaneous edema.

Ultrasound depicted hypoanechoic fluid areas, especially in the lateral quadrants of the left breast, and a diffused ipsilateral parenchymal and cutaneous edema, suggestive of an inflammatory process (Figure 2). 

Considering patient's history and clinical and radiological features, the main diagnostic hypothesis was infective process, foreign bodies reaction, or granulomatous mastitis. 

A subsequent biopsy of the left breast tissue was performed. The pathology report showed chronic mastitis and microabscesses. 

After a month of conservative treatment (antibiotics and steroids), new fluid pockets and chronic suppuration developed. The patient subsequently required a left mastectomy.

Histological analysis revealed multiple areas of inflammatory tissue including fragments of PAS+ amorphous material, suggestive of residues of the pacemaker sheath. 

Foreign bodies granulomatous reaction was the discharge diagnoses.

Four months later, a breast ultrasound was performed; it showed a nonhomogeneous subcutaneous tissue suggesting cellulite. Increased skin thickening and linear fluid pocket upper to the pectoral muscle were associated. 

Few months later, the patient presented with a new unexpected inflammatory symptoms and ulcerated lesions on the contralateral breast. 

She was readmitted to the hospital, and she underwent a digital mammography and breast ultrasound. 

**Figure 3:**
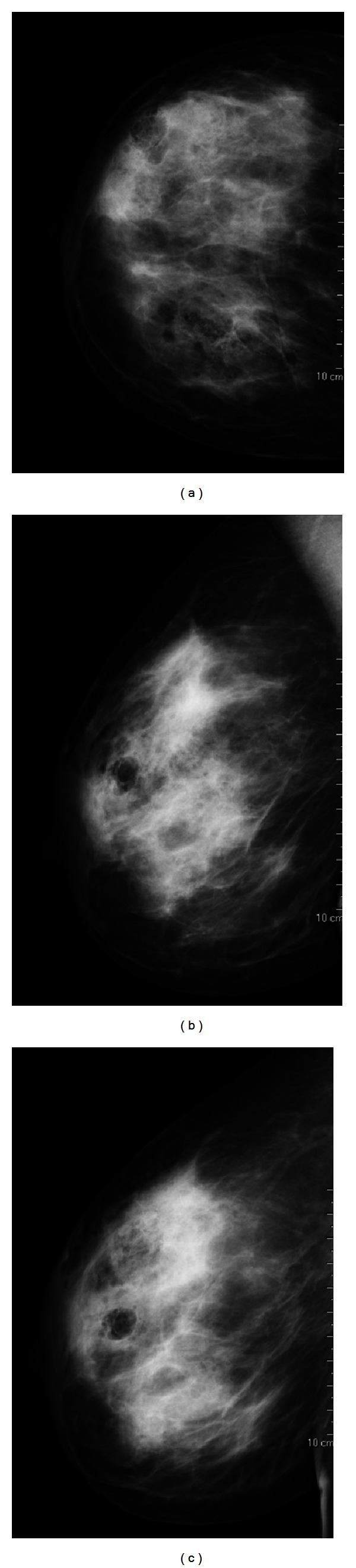
Right craniocaudal projection (a), mediolateral oblique projection (b), and mediolateral projection (c) show diffused increased parenchymal density and increased skin thickening in the areolar region.

Mammography revealed diffuse augmentation of parenchymal density in the right breast with increased skin thickening, in particular in the areolar region (Figure 3).

**Figure 4:**
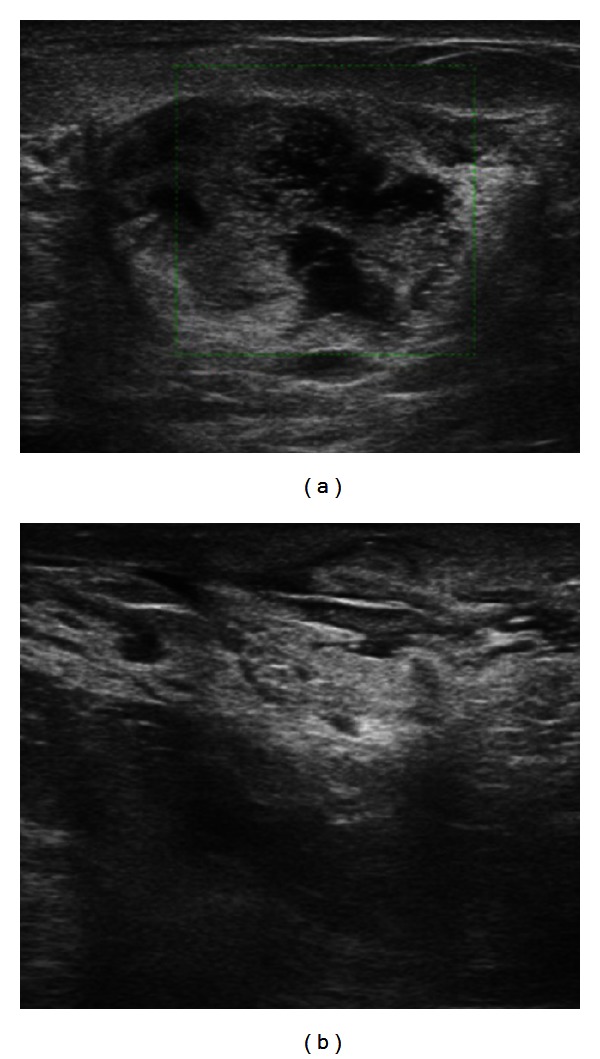
US of the right breast shows a complex heterogeneous mass with hypoechogenicity within, localized in the outer quadrants (a) and a linear hypoechogenicity extending to the skin (fistulous tracts) in the upper inner quadrant and a diffused subcutaneous edema (b).

A complementary ultrasound revealed a complex heterogeneous mass with several areas of hypoechogenicity localized in the outer quadrants of the right breast, suggestive of an inflammatory mass. Ultrasounds also showed a hypoechogenic line extending from the inner inflamed parenchyma to the skin, appearing like fistulous tracts connected with the cutaneous ulcerated lesion in the upper inner quadrant of the right breast. Subcutaneous edema was also depicted (Figure 4). 

Considering those findings, MRI was indicated. Dynamic contrast-enhanced MRI was performed with a 1.5 T unit (Gyroscan Intera, Philips Medical Systems, Best, The Netherlands) equipped with 4 channels reception dedicated coil. MRI images were acquired on axial planes with FFE-T1- and TSE-T2-weighted sequences, followed by dynamic contrast-enhanced sequences. T1-weighted dynamic sequences were acquired previously; 15 mL gadolinium bolus injection (gadopentetic acid and dimeglumine salt, Magnevist; Schering, Berlin, Germany) was administered with a 2 mL/sec flow, followed by a saline flush of 10 mL.

**Figure 5:**
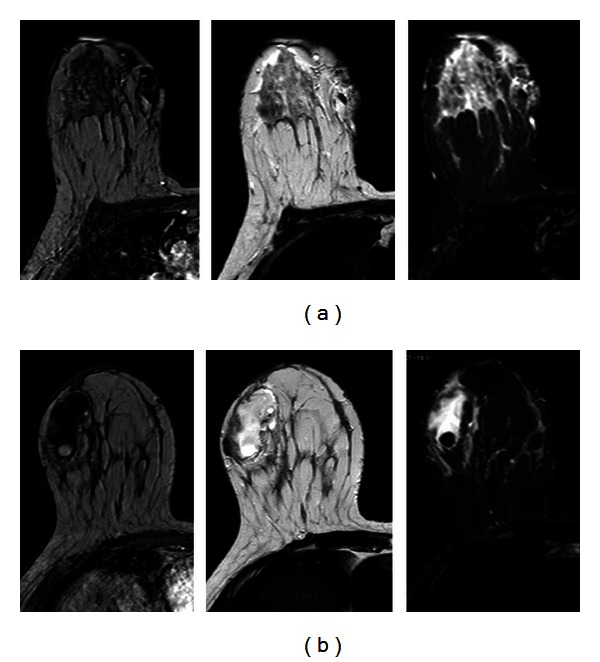
T1-weighted high resolution isotropic volume examination (THRIVE), T2-weighted sequences, and T2-weighted short tau inversion-recovery (STIR) sequences show a structural subcutaneous and cutaneous alteration with pseudonodular appearance in the upper inner quadrant of the right breast (a) and a T1 mixed hyper- and hypointense and T2 hyperintense lesion in the lower outer quadrant of the right breast, suggested for an inflammatory fluid pocket (b).

**Figure 6:**
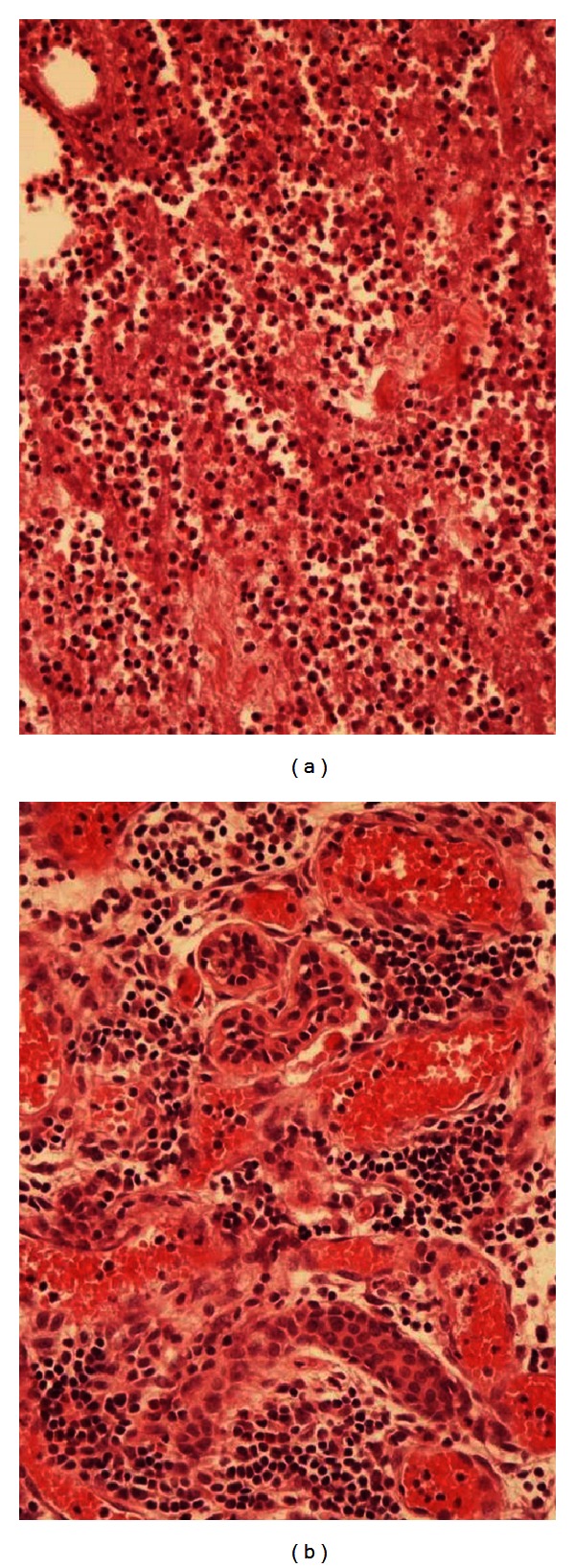
Photomicrograph of the histology: breast tissue was almost completely substituted by necrotic tissue surrounded by heavy polymorph nucleates infiltrate extending from the skin to the adipose tissue. Only few islands of breast tissue were observed (a and b).

MRI showed multiple lesions in the outer quadrants of the right breast appearing both hyper- and hypointense in T1-weighted sequence and hyperintense in T2-weighted sequence. These lesions showed a peripheral ring enhancement in T1-weighted dynamic sequences suggestive of inflammatory fluid pockets. A structural subcutaneous and cutaneous alteration with pseudonodular appearance was also detected in the upper inner quadrant (Figure 5). Biopsy was performed and revealed an extensive necrotic tissue surrounded by heavy polymorph nucleates, extending from the skin to the adipose tissue, suggestive of IGM (Figure 6). The inflammatory tissue presented the same histological characteristics of the inflammatory process that occurred in the contralateral breast four months before, so the first diagnosis of foreign bodies granulomatous reaction was finally changed in IGM. The patient immediately underwent a right mastectomy. Histological findings confirmed the presence of IGM involving breast parenchyma and subcutaneous tissue and skin. 

## 3. Discussion 

Idiopathic chronic granulomatous mastitis, also called granulomatous lobular mastitis or granulomatous lobulitis, is a rare benign inflammatory breast disease of unknown etiology [4]. It was first described in 1972 by Kessler and Wolloch [10]. 

Approximately, 200 cases of IGM have been reported in the literature during the past 3 decades, with most of them being reported in developing countries [11]. 

This disease usually affects women of child-bearing age or those with a history of oral contraceptive use [5]. Mechanisms that have been proposed as etiologic factors include chemical reaction associated with oral contraceptive pills, autoimmune phenomenon, infection with yet unidentified pathogens, and localized immune response to extravasated secretions from lobules. Conditions such as pregnancy, breast feeding, breast trauma, hyperprolactinemia with galactorrhea, and alpha-1-antitrypsin deficiency have been associated with an increased risk of IGM. 

Signs and symptoms typically include a progressive painful breast lump, associated with lesions of variable size, usually firm, tender, ill defined, and unilateral; however, bilateral disease is also reported [3]. The lesions are located in any quadrant of the breast except for the subareolar region [1, 6]. Axillary lymph nodes are usually not enlarged [12].

IGM must be differentiated from other disorders such as inflammatory cancer, infective process, and foreign bodies reaction.

IGM remains a diagnosis of exclusion. Only biopsy and subsequent histological analysis allow a correct diagnosis.

There are no radiological findings that are specific of IGM, but in the appropriate clinical setting, the diagnosis can be suggested by the radiologist. Mammography findings usually include irregular mass and increased parenchymal density; skin thickening and nipple retraction were also noted [4]. Normal mammograms are reported with varying frequency, depending on the size and location of the IGM lesion at the time of diagnosis and the density of the surrounding breast parenchyma [5, 6]. 

Breast ultrasound can depict hypoechoic confluent regions of disease; it is often difficult with this modality to fully delineate the extension of the disease, and the presence of parenchymal or cutaneous edema represents a further limit to the sonographic evaluation. For this reason, MRI is a good imaging examination, allowing a precise and complete delineation of the inflammatory condition, as well as allowing the surveillance of disease evolution. The diagnosis is confirmed by biopsy. 

There is no clear consensus regarding optimal management of IGM. Antibiotics, steroids, and surgery are the options from the least to the most aggressive [13]. Antibiotics are widely used but the most effective noninvasive treatments are corticosteroids. Surgery treatment options are limited or wide local excision and mastectomy. 

## 4. Conclusion 

We reported an unusual presentation of IGM which had bilateral involvement and an unusual progression after mastectomy. Mammography and ultrasound were valid diagnostic tools to delineate the inflammatory disease. MRI allowed a precise extension of the inflammatory condition. MRI was also useful for a correct treatment planning and followup.
